# A Trend Analysis of Condom use in Spanish Young People over the Two Past Decades, 1999–2020

**DOI:** 10.1007/s10461-021-03573-6

**Published:** 2022-01-17

**Authors:** Rafael Ballester-Arnal, Cristina Giménez-García, Estefanía Ruiz-Palomino, Jesús Castro-Calvo, María Dolores Gil-Llario

**Affiliations:** 1 Departamento de Psicología Básica, Clínica y Psicobiología, Universitat Jaume I, Avda. Vicent Sos Baynat s/n, 12071 Castellón, Spain; 2 Departamento de Personalidad, Evaluación y Tratamientos Psicológicos, Universitat de València, Valencia, Spain; 3 Departamento de Psicología Evolutiva y de la Educación, Universitat de València, Valencia, Spain

**Keywords:** Condom use, Trend, Gender, Young people, Spain

## Abstract

HIV exposure is one of the greatest sexual risks in young people, and condom use is the best protective measure. Despite the preventive efforts, trend in condom use is still unclear. This study examines the trend of condom use by gender in Spanish young people, in different sexual practices (vaginal, oral and anal), relationships (regular and casual) and having sex after drugs consumption during the two past decades (from 1999 to 2020). For this, 14,472 people who ranged from 17 to 40 years old (63.5% women) filled the AIDS Prevention Questionnaire in each year. In general, low condom use remains stable and even gets worse regardless of the type of sexual practice, relationship and the substance consumption. Regarding gender, this trend is worse in women who have been less likely to report condom use than men have. Moreover, older people have reported a minor frequency of condom use than the youngest people have done, except for anal sex. Therefore, it is necessary to analyze why, despite preventive efforts, condom use seems to decrease over time.

## Resumen

El VIH es uno de los mayores riesgos en la sexualidad de la juventud, y el uso del preservativo la mejor medida de protección. A pesar de los esfuerzos preventivos, la tendencia en su uso es indeterminada. Este estudio examina la evolución del uso del preservativo según género en jóvenes españoles, en diferentes prácticas sexuales (vaginal, oral y anal), relaciones (estable y esporádica) y al practicar sexo tras consumir drogas, durante las dos últimas décadas (de 1999 a 2020). Para ello, 14.472 personas entre 17 y 40 años (63,5% mujeres) cumplimentaron el Cuestionario de Prevención del Sida cada año. En general, el bajo uso del preservativo se mantiene estable e incluso empeora con independencia del tipo de práctica sexual, relación y consumo de sustancias. Según el género, las mujeres muestran peor tendencia, al informar menor uso que los hombres. Además, las personas más mayores informan menor frecuencia de uso que los más jóvenes, excepto para el sexo anal. Por tanto, es necesario analizar por qué, a pesar de los esfuerzos preventivos, el uso del preservativo parece disminuir con el tiempo.

## Introduction

Sexuality in young people becomes an opportunity to improve their wellbeing but also to expose themselves to various risks. Despite important medical advances, STIs infections such as HIV remains a concern in this population around the world [1, 2]. The high prevalence of HIV-AIDS, together with the stigma that still exists, and the difficulty in testing, increase its severity [1, 3]. This mainly occurs in countries such as Spain, where the unsafe sexual behavior has shown the most worrying trend in HIV transmission over the last decade, and young people account for more than 60% of HIV new infections [4]. As a result, as in other countries, Spanish young people are more affected by HIV than other populations are [1, 2].

In this context, behavioral interventions seem to be the most effective for HIV prevention in the absence of an effective vaccine, even more in view of the improvements that have shown in safe sex behavior, and the non-existence of side effects that may be related to biomedical technologies [5–7]. Consequently, behavioral interventions would become the main challenge to improve this situation. Particularly, inconsistent condom use becomes one of the most relevant risk factors for HIV transmission [8, 9]. In their analysis, some studies have shown how women seem to use a lesser number of condoms [10], even though in some cultures, such as Spain, they have healthier attitudes and beliefs towards HIV [11]. In that sense, men are more consistent with their behaviors when they perceive the risk of HIV infection. This situation seems to be more evident in regular partners, where it has been observed a lesser condom use than in casual partners [12]. Some authors [13] state how the trust and intimacy characteristic of romantic relationships could modulate the perception of HIV risk and, consequently, influence on condom use. In addition, people in regular relationships are more concerned about unintended pregnancy than HIV [14], which facilitates condom substitution by long and short-acting hormonal methods, including the use of intrauterine devices and the hormonal implant, but also oral contraceptive pills, the patch, and the vaginal ring [15, 16]. Perhaps, this could also influence on the fact that vaginal sex is one of the practices in which condoms are used the most, compared to other practices such as oral and anal sex [17–19]. Regardless of the type of sexual practice or partner, alcohol consumption is another factor that increases the exposure to HIV [10]. It decreases the ability to assess risk, as well as decision-making and, consequently, condom use.

In this context, some theoretical models have also analyzed the different variables that facilitate condom use [20, 21]. Beyond highly relevant indicators such as behavioral intention or self-efficacy, past experience with condoms seems to be one of the best predictors of future condom use [22]. Given its relevance, some studies have examined its trend to improve the adjustment of HIV preventive strategies, revealing different results depending on the region. In general, a study conducted in 20 European countries showed how condom use had increased among adolescents from 2002 to 2010 [23]. This result supported a previous study on Portuguese adolescents from 2002 to 2010 [24] in which condom use at last intercourse had increased, although the level of knowledge and attitudes towards HIV had decreased. However, another study focused on Scottish adolescents emphasized a worsening in condom use between 2002 and 2014 [25]. In the United States, between 2002 and 2017, a study [26] reported how condom use during first sexual intercourses had increased among men, but not among women. In this line, a study focused on Canadian adolescents [27], between 2002 and 2014, also showed how men used more condoms while women had frequently replaced them by contraceptive methods. As regards Spain, from 2002 and 2008, a study [28] reported an increase in the use of contraceptive methods among Spanish young people, together with a more inconsistent use of condoms. However, other study among Spanish adolescents [29], between 2006 and 2012, found a higher condom use in the first sexual relationship in men, as well as lower levels of knowledge and attitudes in newer generations. Concerning attitudes, other past Spanish studies [30, 31], had already shown a worsening risk profile for HIV, based on the decrease of perceived susceptibility and condom use intention.

Thus, these results alert us to the importance of deepening the analysis of preventive strategies and behavior, although they do not clearly indicate a trend in condom use at the international level and, even less, in Spanish-speaking countries. In the Spanish context, few studies have analyzed the level of exposure to HIV or, in any case, they have focused on the adolescent population, some specific years and, mainly, socio-cognitive variables which have shown an unsatisfactory trend, with low perceived susceptibility to HIV and a small decrease in the intention to use condoms [32]. Therefore, considering this gap of knowledge and the relevance of condom use for HIV prevention, this study examines the trend of condom use by gender among Spanish young and early adult people, from 1999 to 2020. For this purpose, the analysis makes differences in sexual practices (vaginal, oral and anal sex) and relationships (casual and regular partner), as well as specifies the analysis of having sex after drugs consumption as one of the most important high-risk situations. According to the literature, four hypotheses were formulated:

- Spanish young people will diminish the frequency of condom use over the years regardless of the type of sexual practice, relationship and after substance use.
- In general, Spanish young men will have reported more use of condom than Spanish young women.
- Concerning sexual practices, Spanish young people will have reported more condom use in vaginal sex than in oral and anal sex.
- Concerning the type of relationship, Spanish young people will have reported more condom use in casual partners than in regular partner.
- Additionally, we have asked what role could age play in condom use.

## Methods

### Participants

For this study, 14,472 young people participated. The age ranged between 17 and 40 years old, and its average was 20.85 (SD 3.10). In relation to gender, 63.5% self-identified as women and 36.5% as men. Regarding sexual orientation, 90.5% self-identified as heterosexual, 6.9% as bisexual and 2.5% as homosexual (see Table 1). In addition, 79.4% were having sexual practices at the evaluation moment and 59.1% had a regular relationship.

**Table 1:** Descriptive analyses of socio-demographic characteristics by gender

Socio-demographic variables	Gender		1999	2000	2001	2002	2003	2004	2005	2006	2007	2008	2013	2015	2016	2017	2018	2019	2020
Age M(SD)	Men		20.79 (2.74)	21.64 (3.62)	21.02 (2.62)	20.96 (2.64)	21.17 (2.92)	21.64 (3.24)	21.70 (3.60)	21.48 (3.55)	21.68 (3.59)	20.73 (3.42)	21.12 (3.44)	20.88 (3.45)	21.19 (3.35)	20.88 (3.62)	20.26 (2.75)	20.25 (2.61)	22.87 (3.89)
	Women		20.30 (2.06)	20.52 (2.03)	20.52 (2.35)	20.65 (2.24)	20.83 (2.82)	21.06 (2.70)	21.15 (3.36)	21.11 (3.64)	20.60 (3.32)	20.32 (3.06)	20.78 (3.40)	20.37 (2.80)	20.70 (3.08)	20.53 (3.24)	20.17 (2.49)	19.90 (2.29)	21.78 (3.50)
Sexual orientation (%)	Men	Heterosexual	94.7	92.4	92.6	97	95.1	93.8	92.8	93.1	89.6	93.4	89.2	90.1	88.4	87.2	87.7	88.3	62.6
		Bisexual	3.8	6.3	5.2	2.7	2.9	3.0	3.8	3.1	4.9	3.6	4.5	4.5	5.2	7.6	6.9	5.9	13.2
		Homosexual	1.5	1.3	2.2	0.4	2	3.2	3.4	3.8	5.5	3.0	6.2	5.4	6.1	4.9	5.3	5.3	23.7
	Women	Heterosexual	95.4	95.1	95.8	95.1	97.6	96.6	95.3	92.2	92.2	96.7	93.1	91.4	90.2	86.1	81.2	79.3	69.9
		Bisexual	3.2	3.7	3.6	3.7	1.4	2.7	4.0	6.6	4.9	2.6	4.8	6.0	8.4	10.5	17.1	19.8	27.5
		Homosexual	1.4	1.2	0.6	1.2	0.9	0.7	0.8	1.2	2.8	0.7	2.1	2.6	0.9	2.7	1.8	0.9	2.0
Being in a relationship (%)	Men		51.1	55.6	55	50.6	52.1	54.6	50.2	54.7	51.6	50.4	47.6	40.8	40.1	36.4	38.4	42.5	42.7
	Women		69.4	57.9	64.9	72.7	71.5	75.4	68.8	70.4	67.7	72.5	63.2	49.6	50.1	52.2	53.9	51.6	60.6
Sexual experience (%)	Men		86.6	91.4	93.1	93.6	95.2	93.6	93.0	92.6	95.1	96.2	94.7	92.6	90.4	85.3	87.8	87.8	88.6
	Women		83.6	84.2	88.7	90.2	92.7	96.4	94.8	93.1	95.4	95.5	91.7	91.1	88	88.4	87.8	87.7	90.0

### Instrument

The AIDS Prevention Questionnaire evaluates HIV sexual prevention based on the socio-cognitive models [33–35]. This validated questionnaire includes five factors with adequate internal consistency by 44 items [36]: information, attitudinal beliefs and self-efficacy, behavioral intention, condom use and discrimination towards people living with HIV. For this study, particularly, we have analyzed the frequency of condom use in three types of sexual practices (vaginal sex, oral sex and anal sex), two types of relationships (regular and casual partner), and in a specific risky context (having sex after drugs consumption). For this purpose, participants filled a scale with six Likert items ranging from 0 (never) to 3 (always). This scale obtained an alpha Cronbach of 0.80 for this population.

### Procedure

This study, supported by the Ethic Committee of Research at the university, was developed from 1999 to 2020. During outreach activities about the World AIDS Day located in the University campus, researchers disseminated the information about this study. Participants completed the written questionnaire in 20–30 min individually, anonymously, and voluntarily, when they had given their informed consent. The questionnaire was administered every year, except between 2009 and 2012 and in 2014. The procedure was the same each year, except for 2020. Because of the COVID-19, participants were recruited by the university online networks where we adapted the outreach activities about the World AIDS Day.

**Fig. 1:**
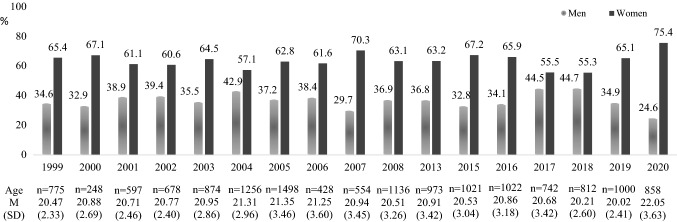
Number of participants by gender and age per year of recruitment

Firstly, 14,618 people were interested in this study but only 14,472 (99% of them) were involved based on inclusion criteria: being from 17 to 40 years old and native-Spanish speaker. The recruitment maintained similar rates of gender and age distribution over the years (see Fig. 1).

### Analyses

The Analyses of variance and the Bonferroni correction were used to explore differences in general and in each gender, from 1999 to 2020. After that, we carried the Student’s t and the Cohen’s d to examine differences by gender in each year for using condoms. Moreover, we carried out the linear regression for all variables to analyze if gender and age were relevant from 1999 to 2020, as well as over the years. For this purpose, we added the interaction analyses between sex*year and age*year.

## Results

### Differential Analyses of Condom use in Sexual Experiences per Year

**Fig. 2:**
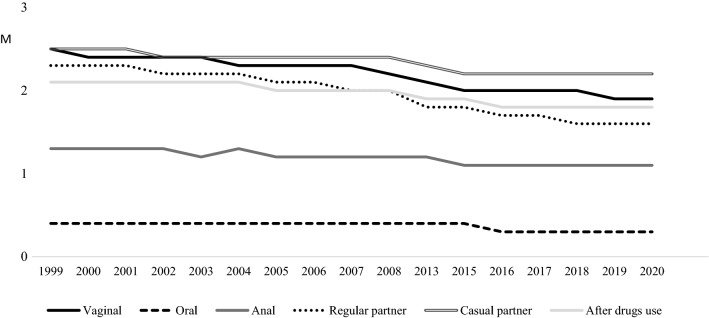
Unstandardized predicted value of condom use in sexual experiences by year

In line with the ANOVA analyses (see Table 2), all practices have revealed statistically significant differences by year in frequency of condom use, which is evaluated between 0 (never) and 3 (always) (see Fig. 2). In case of vaginal sex, the highest mean was in 1999–2000 (M = 2.48) while the lowest was in 2020 (M = 1.91), showing higher means most of the earlier evaluation moments, based on the Bonferroni correction. In relation to oral sex, the highest mean was in 2000 and 2008 (M = 0.56) while the lowest was in 2020 (M = 0.28). In this sexual practice, the lower means were from 2016 to 2020. In case of anal sex, the highest mean was in 2008 (M = 1.61) and the lowest in 2016 (M = 0.91). Based on the Bonferroni correction, means were lower from 2015 to 2018, although these increased in the latest evaluations. About the type of relationship, in the frequency of use of condoms in regular partners, the highest mean was in 1999 (M = 2.38) while the lowest was in 2020 (M = 1.45), being the lower means between 2007 and 2020. In case of casual partners, the highest mean was in 2006 (M = 2.61) while the lowest was in 2019 (M = 2.17), being the lower means from 2015 to 2020. This has also occurred in condom use after drugs that have shown the highest mean in 2004 (M = 2.19) and the lowest in 2018 (M = 1.75).

**Table 2:** Differential analyses of condom use in sexual experiences per year

Condom use	1999 M (SD)	2000 M (SD)	2001 M (SD)	2002 M (SD)	2003 M (SD)	2004 M (SD)	2005 M (SD)	2006 M (SD)	2007 M (SD)	2008 M (SD)
Vaginal sex	2.48 (0.93)	2.48 (0.90)	2.37 (1.01)	2.46 (0.85)	2.44 (0.87)	2.42 (0.92)	2.36 (0.92)	2.38 (0.94)	2.23 (1.01)	2.32 (0.98)
Oral sex	0.48 (0.95)	0.56 (1.03)	0.39 (0.89)	0.42 (0.88)	0.38 (0.80)	0.42 (0.84)	0.44 (0.86)	0.55 (0.97)	0.40 (0.78)	0.56 (0.95)
Anal sex	1.24 (1.37)	1.39 (1.35)	1.18 (1.40)	1.27 (1.30)	1.43 (1.30)	1.23 (1.34)	1.43 (1.36)	1.49 (1.37)	1.14 (1.29)	1.61 (1.28)
Regular partner	2.38 (0.99)	2.36 (1.01)	2.28 (1.07)	2.37 (0.92)	2.24 (1.02)	2.19 (1.04)	2.11 (1.07)	2.17 (1.06)	1.96 (1.16)	2.09 (1.08)
Casual partner	2.32 (1.15)	2.37 (1.13)	2.36 (1.08)	2.55 (0.91)	2.50 (0.97)	2.51 (0.94)	2.51 (0.91)	2.61 (0.85)	2.50 (0.95)	2.47 (0.98)
After drugs use	2.06 (1.13)	2.08 (1.12)	2.07 (1.11)	2.18 (1.06)	2.06 (1.10)	2.19 (1.04)	2.11 (1.06)	2.17 (1.08)	1.98 (1.13)	2.12 (1.07)

Condom use	2013 M (SD)	2015 M (SD)	2016 M (SD)	2017 M (SD)	2018 M (SD)	2019 M (SD)	2020 M (SD)	F(p)	Bonferroni
Vaginal sex	2.17 (1.06)	2.08 (1.09)	2.03 (1.11)	1.95 (1.08)	2.06 (1.01)	2.00 (1.02)	1.91 (1.06)	27.06 (.000)	2007 < 1999 2013 < 1999–2000, 2002–2005 2015, 2018 < 1999–2006, 2008 2016, 2019 < 1999–2008 2017, 2020 < 1999–2013
Oral sex	0.49 (0.88)	0.55 (0.95)	0.35 (0.78)	0.33 (0.75)	0.33 (0.77)	0.38 (0.83)	0.28 (0.68)	6.36 (.000)	1999, 2000, 2005 > 2020 2003 > 2008 2006 > 2016,2018,2020 2008 > 2003,2016–2020 2013 > 2018,2020 2015 > 2016–2020
Anal sex	1.30 (1.30)	1.10 (1.30)	0.91 (1.24)	1.00 (1.27)	1.10 (1.30)	1.16 (1.24)	1.39 (1.26)	5.02 (.000)	2005,2006,2020 > 2016, 2017 2008 > 2015–2019 2016 < 2003,2013
Regular partner	1.80 (1.17)	1.89 (1.16)	1.74 (1.17)	1.71 (1.18)	1.81 (1.13)	1.72 (1.11)	1.45 (1.17)	38.72 (.000)	2005, 2008 < 1999, 2002 2007 < 1999–2004 2013, 2015, 2016 < 1999–2006, 2008 2017, 2019 < 1999–2008 2018 < 1999–2016, 2008 2020 < 1999–2019
Casual partner	2.35 (1.04)	2.19 (1.10)	2.24 (1.10)	2.19 (1.16)	2.24 (1.06)	2.17 (1.07)	2.32 (0.97)	9.43 (.000)	2015- 2019 < 2002–2008 2020 < 2006
After drugs use	2.00 (1.12)	1.89 (1.13)	1.88 (1.14)	1.79 (1.20)	1.75 (1.10)	1.77 (1.11)	1.90 (1.06)	9.58 (.000)	2015,2016 < 2002, 2004–2006, 2008 2017 < 1999, 2001–2006, 2008 2018, 2019 < 1999, 2001–2006,2008, 2013 2020 < 2002, 2004, 2008

### Differential Analyses of Condom use in Sexual Experiences per Year by Gender

**Fig. 3:**
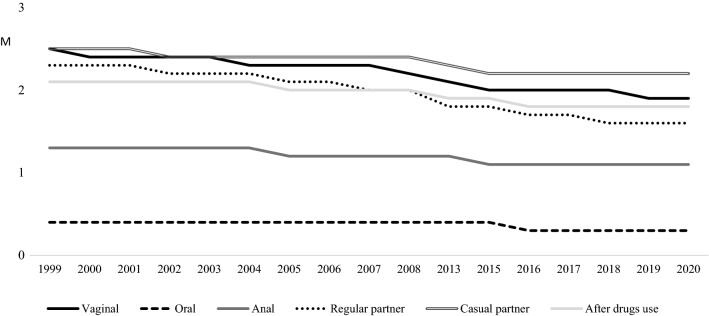
Unstandardized predicted value of condom use in sexual experiences for men by year

**Fig. 4:**
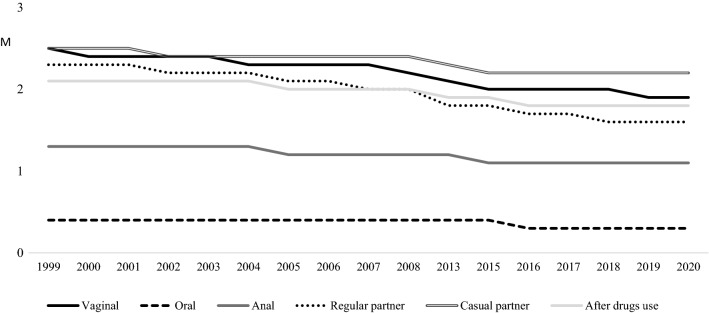
Unstandardized predicted value of condom use in sexual experiences for women by year

In relation to sexual practices in each gender, men and women separately have revealed many statistically significant differences over the years (see Table 3, Figs. 3 and 4), as well as some statistical differences by gender in each year. Particularly, in vaginal sex, the highest mean for men was in 2002 (M = 2.50) while the lowest was in 2020 (M = 1.78). For women, the highest mean was in 2000 (M = 2.53) and the lowest was in 2017 (M = 1.88). In line with the Student’s t, men exceeded women in using condom in 2013, 2016 and 2018, although the effect sizes are low. For both, the lower means have been revealed over the latest evaluations.

**Table 3:** Differential analyses of condom use in vaginal, anal and oral sex per year by gender

Condom use	Sex	1999 M (SD)	2000 M (SD)	2001 M (SD)	2002 M (SD)	2003 M (SD)	2004 M (SD)	2005 M (SD)	2006 M (SD)	2007 M (SD)	2008 M (SD)
Vaginal sex	Men	2.47 (0.88)	2.42 (0.87)	2.32 (1.04)	2.50 (0.80)	2.41 (0.88)	2.39 (0.90)	2.38 (0.89)	2.41 (0.93)	2.10 (1.05)	2.36 (0.95)
	Women	2.48 (0.96)	2.53 (0.90)	2.40 (0.99)	2.43 (0.89)	2.47 (0.87)	2.43 (0.94)	2.34 (0.94)	2.35 (0.94)	2.28 (0.99)	2.29 (1.00)
	t (p)	− 0.12 (.900)	− 0.84 (.401)	− 0.83 (.405)	0.84 (.398)	− 0.87 (.383)	− 0.68 (.491)	0.83 (.406)	0.63 (.528)	− 1.76 (.078)	1.10 (.268)
	d(CI)										
Oral sex	Men	0.38 (0.86)	0.60 (1.12)	0.45 (0.94)	0.52 (0.97)	0.40 (0.86)	0.44 (0.80)	0.41 (0.84)	0.48 (0.92)	0.38 (0.77)	0.51 (0.94)
	Women	0.54 (1.00)	0.55 (0.99)	0.35 (0.84)	0.34 (0.79)	0.36 (0.75)	0.41 (0.87)	0.46 (0.87)	0.60 (1.00)	0.40 (0.78)	0.60 (0.95)
	t (p)	− 1.86 (.063)	0.26 (.788)	1.09 (.273)	1.74 (.083)	0.50 (.614)	0.66 (.508)	− 0.76 (.444)	− 1.02 (.304)	− 0.21 (.831)	− 0.92 (.356)
	d(CI)										
Anal sex	Men	1.28 (1.36)	1.37 (1.33)	1.30 (1.42)	1.52 (1.30)	1.59 (1.27)	1.23 (1.30)	1.62 (1.33)	1.67 (1.38)	1.38 (1.22)	1.76 (1.30)
	Women	1.20 (1.38)	1.44 (1.38)	1.08 (1.38)	1.09 (1.28)	1.20 (1.32)	1.23 (1.39)	1.26 (1.37)	1.36 (1.36)	0.97 (1.32)	1.47 (1.25)
	t (p)	0.43 (.665)	− 0.21 (.832)	0.95 (.339)	1.38 (.171)	1.53 (.128)	0.01 (.991)	2.71 (.007)	1.32 (.186)	1.75 (.083)	1.85 (.066)
	d (CI)							0.26 (0.07;0.45)			

Condom use	Sex	2013 M (SD)	2015 M (SD)	2016 M (SD)	2017 M (SD)	2018 M (SD)	2019 M (SD)	2020 M (SD)	F(p)	Bonferroni
Vaginal sex	Men	2.31 (0.96)	2.04 (1.09)	2.15 (1.06)	2.03 (1.08)	2.26 (0.92)	2.09 (0.96)	1.78 (1.10)	8.51 (.000)	2007 < 2002 2015,2017 < 1999, 2002–2006, 2008 2016 < 1999, 2002 2019 < 1999, 2002–2005 2020 < 1999–2006,2008–2013, 2016,2018
	Women	2.09 (1.10)	2.10 (1.08)	1.96 (1.13)	1.88 (1.08)	1.90 (1.06)	1.95 (1.04)	1.94 (1.05)	21.22 (.000)	2013, 2015 < 1999–2005 2016–2020 < 1999–2008
	t (p)	3.05 (0.002)	− 0.79 (.429)	2.37 (.018)	1.70 (.088)	4.77 (0.000)	1.83 (0.067)	− 1.57 (.116)		
	d(CI)	0.20 (0.07;0.34)		0.17 (0.03;0.31)		0.35 (0.20;0.51)				
Oral sex	Men	0.42 (0.74)	0.45 (0.87)	0.33 (0.75)	0.36 (0.76)	0.34 (0.77)	0.44 (0.88)	0.27 (0.65)	1.47 (.101)	
	Women	0.54 (0.95)	0.60 (0.99)	0.35 (0.79)	0.31 (0.74)	0.32 (0.77)	0.35 (0.80)	0.28 (0.69)	6.40 (.000)	2001,2004 < 2015 2016–2019 < 2008,2015 2020 < 1999, 2006, 2008–2015
	t (p)	− 1.88 (.060)	− 2.30 (.022)	− 0.42 (.675)	0.80 (.424)	0.33 (.739)	1.56 (.117)	− 0.16 (.870)		
	d(CI)		− 0.15 (− 0.29; − 0.01)							
Anal sex	Men	1.42 (1.27)	1.38 (1.30)	1.40 (1.30)	1.37 (1.34)	1.44 (1.34)	1.44 (1.22)	1.85 (1.09)	1.79 (.026)	2020 > 2004
	Women	1.21 (1.32)	0.89 (1.26)	0.56 (1.07)	0.54 (1.01)	0.66 (1.12)	0.96 (1.22)	1.16 (1.28)	6.77 (.000)	2016, 2017 < 1999, 2000, 2004–2006, 2008, 2013, 2020 2018 < 2005, 2006, 2008
	t (p)	1.20 (.230)	3.54 (.000)	6.67 (.000)	6.90 (.000)	4.91 (.000)	3.38 (.001)	4.83 (.000)		
	d (CI)		0.38 (0.16; 0.59)	0.75 (0.50;0.92)	0.70 (0.53;0.87)	0.62 (0.36;0.88)	0.50 (0.27;0.74)	0.56 (0.31; 0.80)

In case of oral sex, both men and women report the lowest means in 2020 (M = 0.27 for men and M = 0.28 for women) while men reported the highest mean in 2000 (M = 0.60) and women in 2006, 2008 and 2015 (M = 0.60). The only statistical differences between both were in 2015, when women exceeded men with a small effect size. In any case, based on the Bonferroni correction, only women report lower means over the latest evaluations.

In case of anal sex, the lowest mean for men was in 2004 (M = 1.23) and the highest was in 2020 (M = 1.85). For women, the highest mean was in 2008 (M = 1.47) and the lowest was in 2017 (M = 0.54). In line with the Bonferroni correction, men and women showed higher means in 2020 although women revealed the lower means from 2016 to 2019. In line with the Student’s t and the effect sizes, men’s means exceeded women’s means in 2005, as well as between 2015 and 2020.

In relation to the frequency of using condoms in regular and casual relationships, each gender has shown statistically significant differences over the years and both men and women have shown the lower means at the latest evaluations (see Table 4). In regular partner, the highest mean for men was in 2002 (M = 2.36) and the lowest was in 2020 (M = 1.36). Similarly, the highest mean for women was in 2000 (M = 2.51) and the lowest was in 2020 (M = 1.48). Based on the Student’s t and small effect sizes, women exceeded men in using condoms in 2000, 2004 and 2007, while men exceeded women in 2013 and 2018.

**Table 4:** Differential analyses of condom use in regular and casual partner, and after drugs consumption per year by gender

Condom use	Sex	1999 M (SD)	2000 M (SD)	2001 M (SD)	2002 M (SD)	2003 M (SD)	2004 M (SD)	2005 M (SD)	2006 M (SD)	2007 M (SD)	2008 M (SD)
Regular partner	Men	2.33 (0.95)	2.15 (1.09)	2.21 (1.09)	2.36 (0.89)	2.17 (1.06)	2.07 (1.07)	2.10 (1.06)	2.20 (1.06)	1.68 (1.22)	2.02 (1.10)
	Women	2.40 (1.01)	2.51 (0.90)	2.31 (1.06)	2.38 (0.94)	2.29 (1.00)	2.28 (1.01)	2.12 (1.07)	2.15 (1.06)	2.08 (1.11)	2.14 (1.06)
	t (p)	− 0.82 (.413)	− 2.12 (.036)	− 0.95 (.338)	− 0.28 (.774)	− 1.50 (.133)	− 3.14 (.002)	− 0.35 (.725)	0.49 (.621)	− 3.26 (.001)	− 1.47 (.140)
	d (CI)		− 0.37 (− 0.69; − 0.04)				− 0.20 (− 0.32; − 0.07)			− 0.34 (− 0.55; − 0.14)	
Casual partner	Men	2.43 (1.06)	2.24 (1.25)	2.36 (1.05)	2.56 (0.85)	2.50 (0.94)	2.45 (0.97)	2.42 (0.94)	2.64 (0.82)	2.36 (1.05)	2.46 (0.98)
	Women	2.26 (1.21)	2.48 (1.02)	2.36 (1.11)	2.54 (0.97)	2.49 (0.99)	2.57 (0.91)	2.58 (0.87)	2.58 (0.88)	2.58 (0.89)	2.48 (0.98)
	t (p)	1.45 (.147)	− 1.12 (.265)	0.04 (.963)	0.25 (.801)	0.05 (.960)	− 1.55 (.119)	− 2.46 (.014)	0.50 (.617)	− 1.94 (.053)	− 0.28 (.780)
	d (CI)							0.17 (− 0.31; − 0.03)			
After drugs use	Men	2.02 (1.13)	1.86 (1.15)	2.05 (1.07)	2.15 (1.08)	2.02 (1.12)	2.07 (1.08)	2.06 (1.06)	2.19 (1.04)	1.77 (1.15)	2.10 (1.08)
	Women	2.09 (1.13)	2.22 (1.10)	2.08 (1.15)	2.19 (1.04)	2.08 (1.08)	2.29 (0.99)	2.14 (1.06)	2.16 (1.10)	2.08 (1.10)	2.14 (1.07)
	t (p)	− 0.61 (.540)	− 1.86 (.064)	− 0.23 (.816)	− 0.40 (.685)	− 0.62 (.531)	− 3.05 (.002)	− 1.01 (.310)	0.24 (.810)	− 2.44 (.015)	− 0.56 (.574)
	d (CI)						− 0.21 (− 0.35; − 0.07)			− 0.27 (− 0.49; − 0.06)	

Condom use	Sex	2013 M (SD)	2015 M (SD)	2016 M (SD)	2017 M (SD)	2018 M (SD)	2019 M (SD)	2020 M (SD)	F(p)	Bonferroni
Regular partner	Men	1.96 (1.09)	1.84 (1.18)	1.80 (1.19)	1.80 (1.19)	2.00 (1.08)	1.79 (1.06)	1.36 (1.17)	9.98 (.000)	2007 < 1999, 2001–2003, 2006 2013 < 1999, 2002 2015 < 1999, 2001, 2002 2016 < 1999, 2001–2003, 2005 2017 < 1999, 2001–2003 2019 < 1999, 2001–2003, 2005 2020 < 1999–2006, 2008–2019
	Women	1.70 (1.21)	1.92 (1.16)	1.72 (1.17)	1.64 (1.17)	1.68 (1.16)	1.67 (1.13)	1.48 (1.16)	32.83 (.000)	2005, 2008 < 1999 2007 < 1999, 2000 2013, 2016,2018 < 1999–2008 2015 < 1999–2004 2017, 2019 < 1999–2008, 2015 2020 < 1999–2013,2015,2016
	t (p)	3.02 (.003)	− 0.99 (.318)	0.99 (.321)	1.65 (.099)	3.47 (.001)	1.46 (.144)	− 1.09 (.275)		
	d (CI)	0.17 (0.02;0.31)				0.28 (0.12;0.44)				
Casual partner	Men	2.39 (0.98)	2.22 (1.02)	2.25 (1.04)	2.13 (1.17)	2.35 (0.95)	2.10 (1.07)	2.23 (0.96)	4.15 (.000)	2015 < 2006 2017 < 2002− 2004, 2006, 2008 2019 < 2002–2006, 2008
	Women	2.33 (1.08)	2.18 (1.14)	2.24 (1.14)	2.26 (1.15)	2.14 (1.14)	2.21 (1.07)	2.34 (0.97)	6.89 (.000)	2005 > 1999 2015 < 2002–2008 2016 < 2004, 2005, 2007 2017 < 2005 2018 < 2002–2008 2019 < 2004–2008
	t (p)	0.71 (.474)	0.47 (.632)	0.04 (.961)	− 1.21 (.226)	2.21 (.027)	− 1.24 (.213)	− 1.20 (.229)		
	d (CI)					0.19 (0.02;0.37)				
After drugs use	Men	2.04 (1.08)	1.92 (1.10)	1.96 (1.10)	1.78 (1.20)	1.94 (1.04)	1.72 (1.10)	1.76 (1.06)	3.09 (.000)	2019 < 2002, 2004–2006, 2008
	Women	1.97 (1.14)	1.88 (1.14)	1.84 (1.16)	1.80 (1.20)	1.60 (1.12)	1.79 (1.11)	1.94 (1.06)	8.78 (.000)	2013, 2020 < 2004 2015 < 2004, 2005, 2008 2016, 2017 < 2002, 2004, 2005, 2008 2018 < 1999–2013,2020 2019 < 2002, 2004–2006, 2008
	t (p)	0.71 (.475)	0.40 (.683)	1.24 (.213)	− 0.23 (.814)	3.54 (.000)	− 0.79 (.426)	− 1.76 (.078)		
	d (CI)					0.30 (0.13;0.48)				

In relation to the frequency of condom use with casual partners, the highest mean for men was in 2006 (M = 2.64) and the lowest in 2019 (M = 2.10). For women, the highest mean of condom use frequency was from 2005 to 2007 (M = 2.58) and the lowest in 2018 (M = 2.14). Regarding gender differences, based the Student’s t and small effect sizes, women exceeded men in 2005 while men exceeded women in 2018.

Finally, in relation to the frequency of using condoms after drugs use, the highest mean for men was in 2006 (M = 2.19) and the lowest in 2019 (M = 1.72), while for women the highest mean was in 2004 (M = 2.29) and the lowest in 2018 (M = 1.60). Gender results have shown statistically significant differences in 2004 and 2007 in which women exceeded men, while men exceeded women in 2018, revealing small effect sizes. Based on the Bonferroni correction, for both the lower means have shown in the latest evaluations, particularly in case of women.

### Linear Regression for Sexual Experiences by Gender, Age, and Year

According to linear regression (see Table 5), the role of year of evaluation seems to be relevant for all sexual experiences. In general, condom use decreases as times goes on, being more notable for regular partner and vaginal sex. This is followed by the age variable, which has been shown to be relevant of four sexual experiences. Specifically, younger people are more related to the use of condoms in vaginal intercourse, regular partner and having sex after consuming substances. On the contrary, older people seem to use condoms more for anal sex. Finally, the gender variable also contributes to the explanation of condom use in vaginal and anal sex. In both cases, men would tend to use it to a greater extent. In any case, explained variances are low and, at most, they reach 6.8%.

**Table 5:** Linear regression: significant coefficients of condom use in sexual experiences by gender, age, year and interactions

Condom use	Variables	B	e	CI	F (p)	r^2^
Vaginal sex	Gender	− 0.07	0.019	− 0.10;− 0.03	190.03 (0.000)	0.045
	Age	− 0.03	0.003	− 0.04;− 0.02		
	Year	− 0.02	0.001	− 0.03;− 0.02		
Oral sex	Year	− 0.005	0.001	− 0.01; − 0.003	6.91 (0.000)	0.002
Anal sex	Gender	− 0.42	0.041	− 0.50; − 0.34	44.11 (0.000)	0.032
	Age	0.01	0.006	0.002;0.03		
	Year	− 0.01	0.003	− 0.02; − 0.01		
Regular partner	Age	− 0.05	0.003	− 0.05; − 0.04	279.65 (0.000)	0.068
	Year	− 0.03	0.002	− 0.04; − 0.03		
Casual partner	Year	− 0.02	0.002	− 0.02;− 0.01	30.44 (0.000)	0.010
After drug use	Age	− 0.01	0.004	− 0.02; − 0.01	43.14 (0.000)	0.014
	Year	− 0.02	0.002	− 0.02; − 0.01		

## Discussion

This study analyzes the condom use among Spanish young people over the past two decades to improve the understanding about sexual risk behaviors and, consequently, the progress and effectiveness of the preventive strategies. Our findings emphasize the unacceptable level of exposure to HIV among Spanish people. Even more, even though the effect sizes and variance explained may be statistically small, in line with our hypothesis the results point a minor use of condom over the past two decades in this population, regardless of the type of sexual practice (vaginal, oral and anal sex), relationship (regular and casual partner) and the evaluated context (having sex after drugs consumption). These results are in line with those studies that emphasized an increase of risky profile for HIV exposure among Spanish people [28] and other populations such as Scottish adolescents [25] or South African young people [37]. However, this worsening differs from past results in other European countries [23], Canada [27] or the United States [26]. This difference could be related to methodological issues such as their focus that was only based on the use of condoms in the first sexual intercourse or the last sexual experience but did not specify if this use was systematic or the type of sexual practice. Moreover, some of these results were conducted before the improvement of the antiretroviral therapy, which may be related to lower preventive behavior [38]. Despite these, there is a high-risk profile in this Spanish population, which could be related to aspects such as the sociocultural factors. Therefore, this specific trend among Spanish people should be analyzed in detail in view of their characteristics. Particularly, if we consider that this decreasing trend of condom use may maintain the growing tendency of HIV in Spain [4].

Firstly, in relation to use of condom in sexual practices and according to past results [18], Spanish people have reported the higher frequency of condom use in vaginal sex, that means using it most of the time, followed by anal and oral sex in which its use was reported sometimes and almost never, respectively. In line with previous results [39], and the role of perception in health models [33–35], due to young people perceive themselves as more exposed to pregnancy rather than HIV, they usually focus on protecting themselves from pregnancy on those sexual practices that may cause it (vaginal sex) by barriers perceived as effective (hormonal contraceptive methods or condoms). In this regard, some studies [16, 40] reveal how a higher use of hormonal contraceptive methods has diminished condom use, as well as reveal less condom use in oral and anal sex than in vaginal sex [17]. Even if vaginal sex seems to be the safest sexual practice, this reveals the more concerning decrease over the years, as other results have already pointed about swingers [19]. Thus, despite the anal sex constitutes an increased risk for HIV infection [41], the low levels of condom use in oral and vaginal sex are a main concern.

About the type of relationship, according to past studies [12, 13], Spanish people have maintained a safer sex behavior in casual partner in comparison to regular relationships. Probably, the greatest fear of pregnancy but not HIV exposure due to the trust and commitment attributed to the regular partner make difficult a higher use of condom [27]. This result is also observed in young populations from other countries such as United States [9, 42] who are less likely to use condoms while they self-perceived in regular relationships. However, as past literature pointed [43–45], young people may consider their relationship as monogamous when may not be the case facilitating the serial monogamy and increasing notably their probability of HIV infection. That is, in the early stages of sexual interaction, people seem not to be very promiscuous but tend to be involve in various shorter romantic relationships that perceived as stable, monogamous, and committed. This type of partners would increase perception of security and diminish preventive measures, influenced by the stereotypes of romantic love and presuming a level of commitment and stability that has often not been agreed. In this context, another risk situation would be related to those people who are unaware about their partner's unfaithful behaviors, engaging in unprotected sexual behavior with them. This phenomenon is particularly concerning in these Spanish people due to using condoms in regular partner reveals the higher decrease.

As regards the sexual context, our study also focuses on having sex after drugs consumption, as it constitutes a high-risk situation [10]. As the literature has shown [46], drugs consumption increases feeling more attractive, as well as the disinhibition to interact socially and sexually with others with whom they would not interact in other circumstances. This disinhibition has also been linked to engage in new sexual practices, multiple partners, and less ability to use barrier methods. In this sense, our findings support a worsening trend over the years, which is particularly worrying based on the important levels of alcohol consumption among the Spanish young people [47]. As past authors reported [37], this phenomenon could diminish their competences to decision-making about their sexual behaviors, as well as their perceived susceptibility that also affects the likelihood of being HIV tested.

Secondly, regarding gender differences, this study has also shown a riskier profile among Spanish women who have reported a lower use of condom over the years, particularly, in anal and having sex after using drugs. In other practices, such as having sex with regular partners, women reported safer sex in the earlier years, but this situation has been reversed in the last decade, in line with the results found in Canadian adolescents [27]. In fact, according to past findings [10, 26], men are more likely to use condoms in practices such as vaginal and anal sex. Therefore, women are more exposed to HIV. Probably, the passive sexual role attributed to women in Spanish speaking countries may difficult an active role of sexual self-care for women, being less likely to manage condom use [48]. Moreover, this inequality becomes more complex by the age gap in romantic relationships, as men tend to be older than women are [9, 49]. Thus, as past literature supported [50, 51], it is essential to adjust intervention to women and include other psychosocial competences, such as assertiveness or self-esteem to empower them in sexual relationships.

Thirdly, our findings widen the focus on a broader age range and demonstrate that both early young and young adult people are at risk of HIV, because of the low use of condoms. In fact, the youngest people have reported more use of condoms in vaginal sex, as well as in regular partner and having sex after using drugs. In line with past studies [9], the use of condoms decreases from the earlier stages of youth to its later stages. Getting older would be associated with being in a regular partner, in which people seem to care more about unwanted pregnancy but not about HIV, increasing the use of hormonal contraceptive methods instead of condoms [16]. Thus, young adults would be at greater risk in vaginal sex and regular partner. This is in line with epidemiology data that points them out as one of the most exposed population [4]. In any case, this study makes a particular difference in anal sex in which older people are more likely to use condom than younger people are. Some authors [52] have emphasized how, in some groups such as men who have sex with men, condom use would be more difficult in anal sex given its stigma. Probably, young adult people would be more competent to manage such stigma and condom use.

At this point, these findings should be considered bearing in mind some limitations. Firstly, the use of a self-reported questionnaire might increase social desirability although this has shown adequate psychometric properties. Secondly, the gap of evaluation makes more difficult to generalize the results, even this study mostly covers the two past decades. Thirdly, despite the large number of participants, there is a lack of sexual diversity representativeness per year and gender. Therefore, including sexual orientation in a new analysis would be essential in the future.

## Conclusion

Beyond these aspects, this study emphasizes a concerning trend among Spanish people regarding HIV exposure in the last two decades. First, condom use in all evaluated sexual practices (vaginal, oral and anal sex) have declined, as well as in having sex with regular and casual partner and after using drugs. Second, this trend seems to be more worsening for women who have revealed less use of condom in some practices and over the years, although men have also shown reduced frequency. Third, based on the frequency of condom use, the youngest people are exposed to HIV but also young adult that most of the time are excluded from preventive policies and campaigns. Even though the low effect size of the year of evaluation and the variance explained indicate that the changes found over time are small, they are statistically significant and point in the direction of lower condom use. In light with these findings, we may assume that past preventive strategies have not been effective enough. Then, future studies should analyze where and why there is this gap. If there is a problem of quantity, the number of preventive actions should be increased. If there is a problem of quality, we should improve our designs and methodologies. In line with scientific knowledge [21, 50, 51], it would be necessary to design participatory interventions to address different variables associated with safer sexual behaviors, those included in socio-cognitive models (such as self-efficacy, susceptibility perceived or perceived barriers of condom use), some psychological variables (such as self-esteem or sexual sensation seeking) and contextual factors (such as alcohol use or the type of partner). Moreover, given the influence of sociocultural characteristics on sexual behaviors, it will also be necessary to consider diversity in gender, sexual orientation, and identity, as well as developmental stage, religious beliefs, or ethnic traditions. Furthermore, these interventions should be comprehensive and include, among other issues, the need to take care of oneself and others in sexual relations. In addition, regardless of the number of sessions, preventive interventions should include reinforcement modules in the medium and long term to support the improvements achieved [5, 6]. To be effective, these interventions also require health trainers with enough knowledge and educational skills to build the confidence of participants [53]. These interventions at the microsystem level should be accompanied by other community measures such as increasing access to barrier methods in different settings, a comprehensive sexual health program in schools and high schools and other community awareness-raising actions that identify HIV prevention and sexuality as a social challenge and not exclusively for certain groups. Thus, in order to improve this situation and diminish the prevalence of HIV new infections, public policies should reinforce their efforts to expand coverage, taking into account the vulnerability of women and the need to maintain programs at all stages of human development. Young adults had not access to the new advances about sexual risk prevention, but neither the younger nor adolescent people have enough access to them now.
